# The Revised Reward Theory of Desire

**DOI:** 10.1007/s10670-025-00932-w

**Published:** 2025-02-04

**Authors:** Jeremy Pober

**Affiliations:** 1 Language, Mind, and Cognition Group, Centre of Philosophy, University of Lisbon, Lisbon, Portugal; 2 Department of Philosophy, Centre for Philosophical Psychology, University of Antwerp, Antwerp, Belgium

## Abstract

I propose and articulate a novel theory of desire, called the Revised Reward Theory. As the name suggests, the theory is based—and expands—on Arpaly and Schroeder’s (2014) Reward Theory of Desire. The initial Reward Theory identifies desires with states of the reward learning system such that for an organism to desire some P is for its reward system to treat P as a reward upon receipt. The Revised Reward Theory identifies desires with a different state of the same system, such that for an organism to desire some P is for its reward system to expect or predict that P will be rewarding (roughly) prior to receipt. The difference amounts to equating desires with what we ultimately find rewarding or satisfying versus those that underlie our motivations to obtain that which we take ourselves to desire.

I argue that the structure of the reward system is incompatible with the original Reward Theory but compatible with the Revised Reward Theory. I demonstrate that this difference has important philosophical implications. I focus on moral responsibility and demonstrate Arpaly and Schroeder’s argument, that addiction can mitigate moral responsibility, turns on this precise difference.

Arpaly, Schroeder, and I all ascribe to a meta-theory called ‘natural kindism’ which identifies mental kinds with neurocognitive kinds. This discussion, in addition to defending a theory of desire, is intended to act as a proof of concept for natural kindism as offering a powerful framework for relating empirical results to philosophical issues.

## Introduction

A commonplace theory of desire is the ‘action theory’—that to desire some p is to be disposed to bring it about that p (e.g. Stalnaker 1984; Smith 1994).^1^ It is sufficiently commonplace that Tim Schroeder (2004, 10) refers to it as “the standard theory” of desire. And there is indeed an intuitive connection between desire and motivation: as Anscombe (2000, 68) put it, “the primitive sign of wanting is trying to get.”^2^

Yet the action theory of desire has come under criticism, motivating many philosophers to seek an alternative view. One influential criticism is Warren Quinn’s (1993) argument that motivation is not sufficient for desiring. Quinn asks us to consider ‘Radioman,’ who is disposed to turn on radios whenever he encounters one. Radioman, as Quinn construes him, gets no pleasure from turning on radios, and can cite no reason *why* he wants to turn on radios: he possesses the disposition to turn them on, and nothing more. It doesn’t seem that Radioman desires to turn radios on, but according to the standard theory, he does.

An approach that might do justice to both Quinn’s argument and Anscombe’s intuition that desire and intentional action have a tight relationship is to identify desires with states that are i) in fact the *typical causes* of intentional action, yet ii) defined in terms that don’t include ‘being the typical cause of an action.’

Arpaly and Schroeder’s Reward Theory of Desire (hereafter, ‘RTD’) is such an approach.^3^ Arpaly and Schroeder define desires in terms of a reward^4^ or reinforcement learning system, which they rightly take to stand in a causal relationship to intentional action.^5^ Desires are identified with states of the reward system (which I will explicate). The reward theory avoids Quinn’s objection to action theories: since simply being disposed to actions promoting a certain goal is insufficient for desire, their theory does not render the verdict that Radioman desires to turn on radios (Arpaly and Schroeder 2014, 152).^6^

Arpaly and Schroeder adhere to a particular philosophical methodology or meta-theory in developing the Reward Theory of Desire, what Schroeder has elsewhere (2006) called ‘natural kindism.’ The basic idea is to take the ‘stereotype’ of a mental kind, understood as a causal role—in the case of desires, the motivation of intentional action—and look to empirical psychology/neuroscience to determine what in the brain plays that role. If the system(s) playing the role are substantively similar across humans, or even across multiple species, then we can say that those psychological states *are* those neural natural kinds (Griffiths 1997).

Arpaly and Schroeder’s (op. cit.) work stands out as an exemplar (along with Prinz [2007]) of using natural kindism to investigate the philosophical consequences of the empirical basis of desires. I focus in this paper on one such case: moral responsibility for addiction.

The reward system constantly computes two values: received reward (at a given moment) and expected reward (at the next moment). Arpaly and Schroeder identify desires with states of the received reward ‘stream’ of the system. As Arpaly and Schroeder rightly note, the neuroadaptations in chronic addiction occur in the *expected* reward stream: in particular, addicts’ reward systems consistently over-predict how much reward their drug of choice will bring (Berridge and Robinson 1998).^7^ Because addiction affects not our desires themselves but a low-level prediction (Arpaly and Schroeder 2014, 124), addictive behavior does not, they claim, issue from our intrinsic desires. But being issued from our intrinsic desires is (per their view) what makes us responsible for our actions.

However, I will argue here that desires cannot be states of the received reward stream, but instead must be states of the expected reward stream. I will articulate the details of a view, which I call, the Revised Reward Theory, in which this is the case. The difference amounts to identifying desires with what we take (in the right way) to be rewarding rather than what we ultimately find rewarding.

Setting the table—and in particular describing the reward system’s function in sufficient detail—will take us through Sect. 3. In Sects. 4–7, I will argue for the adoption of the Revised Reward Theory.

While this paper is about desires, it also serves as proof of concept for natural kindism. Natural kindism offers a framework to show how empirical findings can be incorporated fruitfully into philosophical discussion. And the way that moral responsibility for addictive behavior changes based on how the reward system is organized, which I discuss in Sect. 8, demonstrates exactly how this framework plays out in practice.

## Natural Kindism

Natural kindism is a meta-theoretical position about how to *generally* relate empirical findings in psychology and cognitive neuroscience to philosophical issues, and natural kindism is a (promising) answer. What came to be called natural kindism originated in the philosophy of emotions literature, where Griffiths (1997) used it to develop an account of emotion types like ‘fear’ (or ‘schadenfreude’). Natural kindism has gained traction both within the emotions literature in which it originated (Prinz 2004; Scarantino 2012; Pober 2018; Kurth 2019) and in philosophical psychology more generally (Zachar 2000; Perez 2004; Machery 2005; Samuels 2009; Michaelian 2011; Pober 2013; Kumar 2015; Cheng and Werning 2016; Gomez-Lavin 2020; Taylor 2020).

Griffiths (1997) conceived of natural kindism as a development of causal theories of meaning and natural kinds (Kripke 1972; Putnam 1975).^8^ He starts with Putnam’s (Ibid.) notion of a ‘stereotype’ of a natural kind term, that is, properties we already associate with its meaning. For mental states, the stereotype is a causal role, much along the lines suggested by Lewis (1966). Natural kindism looks for the physical basis of the casual role specified by the stereotype. If the causal basis is a natural kind—in the case of psychological states, a neural kind at some level of description—then we identify the psychological kind with the neural kind.^9^ However, if there is no such neural kind, then we remove the mental concept from our (scientific/explanatory) vocabulary.

‘Identify’ should be understood loosely. The relation between the psychological and neural kind can be understood reductively, identifying psychological states with their neural realizers (as in Kim 1998) or non-reductively, taking psychological kinds to be numerically distinct from their realizers (as in Antony 2008). For further discussion, see Shea (2013). On both views, the neurocognitive systems which realize psychological kinds—like the reward system—are themselves functionally individuated. Natural kindism is thus no threat to functionalism of the reductive or nonreductive variety.

Griffiths (1997) claims that instances of ‘basic’ emotions like anger in humans and other mammals just are states of an ‘affect program’ because an affect program is the causal basis of the stereotypical effects of anger (increased heart rate, adrenaline, stereotypical facial expressions, etc.). Affect programs are functionally individuated, neurally realized kinds. However, Griffiths argues that because different emotion types have very different neural bases—some (the so-called ‘basic’ emotions) are affect programs whereas other (‘higher’ emotions) involve higher cognitive mechanisms—emotion is at best two kinds. And when a term intended to be a natural kind ends up referring to a conjunction of two, it, like jade (Kim 1992), must be eliminated.

The upshot is that, if some neural kind is identified with/as the realizer of a psychological kind like ‘belief,’ the properties of the neural kind determine—indeed, *are*—the properties of beliefs (in the relevant species set) in addition to those determined by the stereotype. In this way, natural kindism offers a basic conceptual framework for relating empirical results to philosophical theorizing. As we will see throughout the discussion, using the case of desire, the additional, empirically determined properties matter for downstream philosophical issues.

Finally, natural kindism does not require any particular theory of natural kinds. Most natural kindists listed above, employ a version of the “Homeostatic Property Cluster” theory of natural kinds (Boyd 1991). However, Arpaly and Schroeder do not commit to any particular theory of natural kinds. All they are committed to for a natural kind “is that it is a kind that scientists find useful, at the relevant level of investigation, for correct explanation, prediction, and control of psychological phenomena” (Arpaly and Schroeder 2014, 127n2).^10^

## Reward and the Reward Theory of Desire

The first step in explicating Arpaly and Schroeder’s Reward Theory of Desire is to determine what sort of desires are the *explanans* for their theory.

### Preliminaries

Arpaly and Schroeder distinguish between intrinsic, instrumental, and realizer desires. Both instrumental and realizer desires can be best understood in comparison to intrinsic desires: the intrinsic/instrumental distinction is the familiar distinction between desiring some P for its own sake or desiring some P for the sake of some R, and the intrinsic/realizer distinction is the distinction between desiring some kind of P, and desiring some object that is a token of type P. They aim to give an account of intrinsic desires, as “instrumental and realizer desires do not appear to have interesting lives of their own: they are mere manifestations of a person’s intrinsic desires [combined with their] beliefs” (Arpaly and Schroeder 2014, 9). Given this focus, I hereafter use ‘desire’ to refer to intrinsic desire unless otherwise noted.

They further distinguish between standing and occurrent desires. Standing desires are the sort of desire one can possess while “asleep, anesthetized, and the like” (Schroeder 2004, 134). Whereas occurrent desires are momentary states that motivate action at a time. For example, the mental state that actually gets an agent to procure and eat some cake must be an occurrent desire (or a series thereof). In what follows I will use ‘desire’ to refer to standing desire and specify when I am discussing the occurrent variety.

### Desire as Reward

The Reward Theory is:

R: To have an intrinsic desire regarding it being the case that P is to constitute P as a reward. (Arpaly and Schroeder 2014, 127).

Reward here is used in a technical sense such that “what makes something a reward is that it triggers a specific sort of learning” (Ibid., 61). The type of learning Schroeder alluded to above is *reinforcement* learning. Reinforcement learning is a procedure that underlies “the sort of learning we refer to when we say ‘he learned to speak more in class’” (Arpaly and Schroeder 2014, 130). This technical definition of ‘reward’ which Arpaly and Schroeder, is what I will mean by ‘reward’ unless otherwise specified.^11^ The technical sense is related but not reducible to the colloquial sense of reward: e.g., obtaining a ‘technical reward’ does not require effort, and one cannot fail to notice that one received a ‘technical reward’ (Schroeder 2004; 60).

### Introducing the Reward System

The reinforcement learning—hereafter, for simplicity, ‘reward’—system is defined functionally (for its inputs and outputs) and formally (for the transformations between them) as follows (description paraphrases Sutton and Barto 1998). The reward system makes two computations based on its proprietary inputs. First, it takes as input representations of an organism’s environment in any cognitive or perceptual format (Schroeder 2004, 49) and, from them computes the reward in the environment at a time t_0_. The total reward in an environment at a time is the sum of the reward value of the objects in the environment. Reward systems have values recorded for object types that have been encountered: this is the body of information that is updated in reinforcement learning. The reward system then calculates, based on this information plus probabilities of obtaining various rewards (Schultz 2016) as reward *that an organism can expect to receive* in the near future t_1_. Simultaneously—and also continuously—it takes from the same input sources plus interoceptive representations to perform its second computation, which determines the amount of reward the organism has received/is receiving at t_0_.

The system then compares the two computed values—expected and received reward, respectively—and produces an *error prediction* signal. This signal is calculated by subtracting actual reward at t_0_ from the amount of reward expected or predicted for time t_0_, which was predicted *at* time t_-1_.

This error prediction signal is the basis for learning. It can generate a positive value when amount of reward received exceeds expectations, or a negative one when it fails to meet them. These signals then update the reward values of the objects in the environment that either failed to meet or exceeded expectations. The output of the system is to reinforce (increase) or extinguish (decrease) the reward value of an object. Doing so has the effect that the behavior that caused the organism to obtain the reward is more or less likely to be performed in the future in the presence of a token instance of that kind of reward.

### Desire as Reward, Redux

To illustrate how this all fits together, Arpaly and Schroeder give the example of Juan, an inexperienced dancer dancing with a more experienced partner. In this story, Juan “perceives the approach of his partner and … [performs] a matching step backward on his part. Suppose that what follows this step is that Juan’s partner smiles and the dance continues smoothly. This smile or smooth dancing (or what it signifies) might be a … reward … in the sense relevant to reward… learning … [i]f Juan’s brain responds by releasing a positive learning signal” (Arpaly and Schroeder 2014, 131).

Assuming Juan’s brain does respond by releasing a positive learning signal, The Reward Theory makes the following claims about Juan’s desires. First, he desires, in some sense, his partner to smile. This desire is plausibly a realizer desire for something signified by that smile, such as his partner’s approval, for which he has an intrinsic desire.

### States of the Reward System

The Reward Theory not only specifies what it is *to* desire that P, it also provides an account of what *a* token desire that P is. This latter aspect is required for the Reward Theory to count as a natural kindist view, since desires, *qua* states, must be identified with *states of* the reward system. One option, that Arpaly and Schroeder rightly reject, is to identify a desire that P with the momentary activation of the reward system that constitutes a token positive learning signal associated with P. A momentary activation might be a good account of an occurrent desire, but not a standing one.

For this reason, Schroeder claims “desires … need not involve actual episodes of representing [the object desired] … Rather, to desire is to be so organized that tokened representations of [the object desired] … if they occur, will contribute to the production of reward signals” (Schroeder 2004, 134). Notably, this type of standing state isn’t itself a dispositional state, though it might be the causal basis of one.^12^ It is a categorical state, albeit one which typically lasts much longer than occurrent desires do. More precisely, it is an *organizational* state of the reward system: a way the pieces of the system are linked together. We can analogize such a state to a light bulb with a switch, in which the striking of a switch (the entokening of a representation of a rewarding stimulus) can turn on the light bulb (produce a learning signal). Desires, per Schroeder (Ibid.), are the light bulbs.

### The Reward System as a Natural Kind

The reward system as described is clearly a functional kind: it has proprietary inputs and outputs as well as a formal characterization of how the transformation between them is achieved. It is also, in humans (and mammals more generally) realized in the ‘midbrain dopamine’ circuit, a neural kind. More specifically, Schroeder (2004) identifies the neural basis of the reward system with a circuit comprised of the ventral tegmental area, substantia pars nigra, and basal ganglia (Schroeder 2004, 50, 116). Per Schroeder, the ganglia is involved in both reward computation and production of movement, (Ibid., 116), and the other neural realizers of the reward system output to crucial systems that initiate action, including the motor cortex and supplemental motor area (SMA), plus motor regions of the anterior cingulate cortex (ACC) and prefrontal cortex (PFC) (Ibid., 110, 113). However, whether the systems Schroeder identifies do in fact compute reward has been called into question (Berridge 2012; Jeong et al. 2022). To my knowledge, the idea that reward values are processed—and predicted—has not been challenged: the error prediction model is still safe.

## Nagging Doubts

Consider Marge, who has never tasted frog legs. She believes she will hate them and is grossed out by the mere thought of eating them. However, at a particular dinner party where frog legs are being served, she decides to give them a try in order not to upset her host. She keeps an open mind about the smell and presentation as the course is brought out. She takes a bite, and, as it turns out, she loves the taste, and happily eats the rest. On later occasions, she orders frog legs when they are available.^13^

Next, consider Homer. He remembers loving Milky Way candy bars in his childhood. As an adult, he buys one on a whim one day. Seeing the wrapper makes him think imagine the pleasure he had as a child eating (likely far too many) Milky Ways, and his mouth starts watering. When he bites into it, he expects the same level of pleasure he derived decades ago. As he unwraps the candy, he feels his mouth watering. But when he bites into it, he finds it altogether too sweet, with no complexity to its flavor.

A plausible interpretation of Homer is that his reward system predicted a positive reward value for the Milky Way bar, but he received no reward; his reward system generated a negative learning signal. Marge, on the other hand, plausibly expected no reward from the idea or presentation of frog legs, but did in fact receive reward; her reward system generated a positive learning signal.

Per the Reward Theory, Marge in fact desired frog legs all along, whereas Homer had not in fact desired a Milky Way since childhood. For Marge’s reward system recorded a positive learning signal, and thus by entailment of their view, Marge desires whatever it is that created that reward signal. Homer, on the other hand, no matter how much he *seemed* as an adult to desire a Milky Way, did not, since his reward system generated a negative, rather than positive, signal upon receipt of the Milky Way.

These conclusions strike me as quite incorrect. Nonetheless, they are hardly fatal objections to the Reward Theory. Rather, they put the burden on it to give a plausible story about what desires *did* motivate Homer to act and Marge feel disgust, but it can do so. Here’s how: Marge, who was rewarded by eating frog’s legs although she expected to hate them, did not, unbeknownst to her, always intrinsically desire frog legs, but instead desired something like [things that taste like chicken], and simply lacked the (true) belief that frog legs taste like chicken. That is, Marge she simply had false beliefs about the extension of her extant desires. Likewise, Homer had the desire for a certain taste experience along with a false belief that the candy which provided that experience when he was a child would likewise provide it as an adult.^14^

In both cases, the actual representation that triggered (or failed to trigger) reward learning was not obvious to the person whose mind contained the representation in the first place. In other words, they lacked self-knowledge of the true contents of their desires. Given the range of representations that can trigger the reward system and thus act as parts of desires, it should be no surprise that some kinds will be below the level of conscious awareness. Consequently, as Schroeder (2004, 98) says, “it is no great innovation to discover desires where none were thought to exist before.”

Something still feels amiss, though. While the Reward Theory certainly gives a story of Marge and Homer’s desires, it doesn’t give the story of the desires we intuitively think they have. What strikes me as the most straightforward interpretation of Marge and Homer’s respective behavior is that Marge did not antecedently have, but formed, a desire for frog legs, and that Homer very much desired that Milky Way.

What is really going on is that our (or at least my) intuitions want to attribute to Homer and Marge desires whose content lines up with Marge and Homer’s *expected reward streams*.

This intuition is worth exploring as a starting point for a theory. In the rest of this paper, I will propose my Revised Reward Theory, in which desires are states of the expected reward stream, and discuss its implications. This Revised Reward theory, while motivated by the intuitions discussed in this section, in no way turns on them.

## The Revised Reward Theory

In order to make my case, I will first need to articulate the Revised Reward theory, including specifying the alternative state of the reward system it identifies with desires. Doing so will require a bit of further exposition of the reward system.

### The Reward System: Conditioned and Unconditioned Stimuli

The reward system can be understood as attempting to predict reward as far out in the future as possible.^15^ The further out a prediction, the more valuable it is. To this end, when the reward system finds that an antecedently neutral stimulus is associated with a rewarding one but appears first, it uses that stimulus as a *predictive cue*, responding in part or whole to the cue rather than the reward itself. It learns the predictive value of such a cue through multiple trials: it does not want to mistake an accidental association for a substantive one and act on a bad predictor. Thus these predictive cues are also called *conditioned stimuli*, in contrast to directly rewarding *unconditioned* stimuli. Note that conditioned stimuli should not be equated with instrumental desires: conditioned stimuli are more like indicators of standing desires, whether those desires are intrinsic, instrumental, or realizer.^16^ Nonetheless, conditioned stimuli often cause reward signals *in place of* the stimuli which they indicate. They can indicate either further conditioned stimuli (in which case they are higher-order) or unconditioned.

Consider the following experiment by Schultz et al. (1992). Monkeys are placed in a room with two levers. In the room, the monkeys receive a reward—a cup of apple juice—if they press the left, but not the right, lever upon the presentation of a blinking light. During early trials, the monkeys “behave[d] somewhat erratically” (Glimcher 2011, 15,650), pressing the right and left levers, and the midbrain dopamine system—the physical basis of the reward system—only increased in neural activity that corresponded with the generation of a learning signal (‘spiked’, for how the activity looks on an EEG) upon receipt of the juice reward. Later in the task, however, the monkeys exclusively press the left lever on presentation of the start cue, and the learning signal is generated upon presentation of the blinking light and *not* the obtainment of the reward.

To understand this data, we need to invoke a distinction between two types of events that can generate a learning signal in virtue of having expectations exceeded: a distinction which requires the conceptual apparatus of conditioned and unconditioned stimuli to grasp. Specifically, we must distinguish between the unexpected reward value of an unconditioned stimulus, and the introduction of a conditioned stimulus or cue into an organism’s perceptual field (Schultz 2016). In the former case, the mismatch between expectations and reward is that, while the organism is cognizant of what is in its environment or perceptual field, it does not know—and underestimates^17^—how rewarding (one of) those things actually is. In the latter case, something that is or indicates reward enters the organism’s perceptual field, where before it had been absent. In this scenario, expectations are exceeded in a different sense: the reward system increased its expectations upon the introduction of the cue. There was no mismatch between expected and received reward; rather, there was a mismatch between expectations at t_0_ and t_1_.

Returning to the Schultz et al.: the juice acts as the unconditioned stimulus. Call the beginning of a trial t_0_ and the moment when the monkey drinks the juice t_+1_. Let us idealize momentarily and suppose that the monkeys had never tasted juice before. Consequently, in the first trial, there were no predictive cues that could possibly suggest the presence of a reward by indicating the presence of juice: juice was not yet known by the monkey to be rewarding. Thus, the ‘spike’ indicating an error/prediction and learning signal corresponds with activity in the received reward stream at t_+1_, i.e., on receipt of the juice. No increase in reward was ever *predicted* before it was received.

The actual experimental data for early trials is a bit more complex than this idealized story would indicate. The results showed that the monkeys generated a learning signal upon *receipt* of the juice, not *consumption* of the juice. And it is consumption that is the true rewarding stimulus (Berridge and Robinson 1998; Wise 2004; 2009). What is likely going on is that the *receipt* of the juice was already acting as a predictive cue for the *drinking* of the juice. This result is what one would predict if the monkeys had all at some point tasted juice before participating in the experiment. Wise notes that representations of perceptual properties of rewards can often act as predictive conditioned stimuli. In his words, after the yellow skin of a banana “has been associated with the taste and post-ingestive consequences of the fruit, it becomes a … learned predictor [sic] of reward” (Wise 2004, 486).

In any case, the monkeys learned by the later trials that the blinking light which indicated that juice was about to be obtainable. At that point, they knew what to do by the time they saw the light. The light thus became a conditioned stimulus such that the monkey now ‘expects’ the juice whenever it sees the blinking light, and, when it drinks the juice, there is a reward, but there is no longer an *unexpected* reward (of course, the perception of the juice was a conditioned stimulus as well, but the light was a better one, insofar as it appeared first). At t-1 the monkey was predicting no reward, but as soon as the conditioned stimulus came into sight at t0, the value of the predicted reward stream increased and a learning signal was generated. The ‘error’ prediction reflected a change in the environment, not an actual error on the monkey’s part. When the juice was obtained at t_1_ or consumed at t_2_ there was no activity on receipt of the reward because there was nothing to learn: the monkey already knew everything it needed to.

### A Revised Reward Theory

As I noted at the end of the previous section, the basic idea behind the Revised Reward Theory is that desires should line up with expected, rather than received rewards. Now, we can tentatively formulate the Revised Reward Theory as the conjunction of:

1. To desire that P is to expect (in a certain way) that P will be rewarding, and
2. A desire that P should be identified with the standing state of the reward system to treat representations of Q’s as the basis for a learning signal iff Q’s are conditioned stimuli (predictive cues) for P’s (unconditioned/directly rewarding stimuli).

Thus, the relevant state of the reward system is one that responds to predictive cues: a monkey desires juice just in case its reward system responds to the presence of indicators of juice.

As in the initial Reward Theory, we desire the set of entities whose obtainment/consumption is unconditionally rewarding, with the exception that it is only those phenomena whose representations are also—already—paired with predictive cues. This leaves out very few unconditioned stimuli—only those we have no idea we will like, as Marge thought about her frog legs. And like their view, it is cognitive or perceptual representations that form this input. But unlike Arpaly and Schroeder’s view, it is not the standing state to treat P as the basis for a learning signal that token-identifies with a desire that P, rather, it is the standing state to treat some Q that is associated with P as the basis for a learning signal that token-identifies with a desire that P. The association between P and Q is therefore required to exist in the brain: if there is no extant, real connection between P and Q, then it makes no sense for a person’s desire that P be identified with the state of the reward system that responds to representations of Q. This change carries the following implications:

- For the ontology of desires: if we want to include representational capacities as proper parts, as Schroeder suggests, then the proper parts would have to include both representational capacities–those underlying representations that p *and* representations that q—as well as the associative link between them.^18^
- For desires at the level of persons: to identify desires with states of the expected reward stream, as I do, renders the contents of desires as what we take—in a certain way—to be rewarding or predict will be rewarding. Whereas identifying desires with states of the received reward stream, as Arpaly and Schroeder do, is to identify the contents of our desires with what we will ultimately find rewarding.
- There will also be neural implications, however, I refrain from discussing the neural realizers of the system I’ve here described functionally. I make this omission although I endorse the way in which natural kindism invokes neural states, I set the topic aside until the issue mentioned at the end of Sect. 3 has been settled.

### An Amendment

The Marge and Homer examples from the previous section were described so that they would do ‘double duty.’ In addition to motivating the case for the Revised view, they also were described in a way that demonstrate the existence of two types of expectations. The way I set up the examples, Marge’s expectations about frog legs was belief-based, whereas Homer’s expectations were at a lower level and expressed in things like his salivating. Arpaly and Schroeder distinguish between ‘intellectual’ and ‘visceral’ expectations (Arpaly and Schroeder 2014, 134; Schroeder 2004, 96, wherein he refers to them as ‘gut-level’ expectations). Intellectual expectations are those that are represented propositionally and are the content of beliefs. Visceral expectations are affective states that Schroeder (2001; 2004) takes to be conscious manifestations of the activity of the expected reward stream. ‘Visceral expectations’ are thus a bit of a misnomer: the expectations themselves are not visceral, rather, they are expectations computed by the reward system. And the visceral or affective state is not an expectation: it is an indication of one.^19^

Nonetheless, Arpaly and Schroeder are right to point out that the expectations computed by the reward system—which are, on my revised RTD, token-identical to desires—are distinct from cognitive expectations. The upshot for the current discussion is that when I claim to desire that P is to expect, *in a certain way*, P to be rewarding. I can now say that “this certain way” is via the expected reward steam. We can thus replace 1) from above with the final formula:

1a) To desire some P is to expect, via the organism’s expected reward stream, that P will be rewarding.

## Occurrent Desires on the Reward Theory

Here, I will show that *if* desires are states of the reward system, then they *must be* states of the expected rather than received reward stream. I take that antecedent to be satisfied. I agree with Arpaly and Schroeder that the reward system is the “natural psychological kind … [that] plays all of the causal roles that intrinsic desires play” (Arpaly and Schroeder 2014, 143) and that consequently, “intrinsic desires are states of” it (Ibid.).

It is curious that Arpaly and Schroeder do not further pursue the question of causal role when it comes to which states of the reward system are causing what desires cause. After all, it is not the reward system itself but some state of it that will be identified with desires. And I will show that once we raise this question, it will be states of the expected rather than received reward stream that do the causal work of motivating action through giving rise to occurrent desires. Thus, while both are accounts of standing desires, only the Revised Theory can offer an account of how standing desires relate to occurrent ones.

The most prototypical aspect of the causal role of desire is to motivate intentional action which, harkening back to Anscombe (2000, 68), can be described as “trying to get.” This type of action has to occur before the object of desire is obtained/realized. But the received reward stream is recording the amount of reward the organism is receiving in the present, not the future: it records the reward value of an object when that object is obtained, not prior to its being so. The expected reward stream, however, records the value of a stimulus that is predicted to be rewarding as soon as its impending obtainment enters the mind of the organism, like when the light goes on after the monkey presses the correct bar.

Consequently, any *potential activity* in the expected reward stream with respect to a given stimulus will occur before its receipt (at latest upon its receipt if consumption is the real reward), whereas any potential activity in the received reward stream must occur after receipt or consumption. Or, more precisely: both the expected and received reward stream are always active. By ‘activity’ I mean the activation, change in activity level, or ‘spike’ in neural activity. By the timing of ‘potential activity,’ I mean the time at which activity in the relevant reward stream, if there were to be any, would occur. It is therefore the expected reward stream that is temporally situated in the right place to play the causal role of desires in producing activity.

This argument is sound—as applied to *activity* of the reward streams. Yet the activity of the reward system will correspond to occurrent desires, and Arpaly and Schroeder are giving an account of standing desires, not occurrent ones. Nonetheless, we need to take a closer look at how standing and occurrent desires should be expected to relate to each other. Doing so will make clear why the state they adopt is the wrong one.

Standing desires are organizational states that include representational capacities. They are activated—like a light bulb being switched on—via token representations. Standing organizational states that are waiting to be activated have occurrent states as their token activations. Occurrent desires are the reward system actively responding (in the right way) to entokened activating representations, arguably plus the activating representation as a proper part (I remain neutral on the mereology). The organizational states are like a light bulb and its occurrent counterparts are the light bulb being turned on: the latter just are the activity of the former.

A standing state of either reward stream can only generate its occurrent counterpart when it is capable of being active. And, with respect to a given desire that P, only the expected reward stream can be active at the right time. Occurrent desires thus must be token activations of the expected reward stream. But given the relationship implied between occurrent and standing desires by this model, standing desires must also therefore be states of the expected reward stream.

## Desires, Representation, and Direction of Fit

Arpaly and Schroeder are aware of the possibility of identifying desires with states of the expected reward stream and give their reasons for rejecting it. In this section, I attempt to assuage their worries. I believe that their rejection of the Revised view is motivated by a conception of what desires must be like, and I argue that this conception is misconceived.

The expected reward stream is a type of prediction—a prediction about how much reward the organism will receive in the very near (perceivable) future—and predictions are representations. For they bear content regarding a state of affairs that has not yet come to pass. Consequently, Arpaly and Schroeder claim: “[i]nsofar as it is correct to treat it as a predictive system, it is impossible to see it as instantiating desires. Predictions are true or false; they make claims about how the world will be. Desires are neither true nor false, and they make no [such] claims” (Arpaly and Schroeder 2014, 286).

For Schroeder, having a truth value is a property of representations *as such:* in his words (2004, 65), “one of the characteristic features of representational systems is precisely that they have the capacity to misrepresent the world.” And indeed, in Arpaly and Schroeder’s picture, desires are not identified with a system that represents anything. The received reward stream cannot misrepresent—and therefore cannot represent generally—how much reward an organism is obtaining at a given moment because it is *constitutive of* how much reward an organism is receiving at a given moment.

It is true that the received reward stream does not represent (though it does contain information). But why can’t *desires* represent? Indeed, given that they involve representational states as proper parts, *how* can they not represent? Arpaly and Schroeder endorse a ‘direction of fit’ (Searle 1983) conception which carries this implication.

According to this picture, beliefs (and other cognitive states) have ‘world-to-mind’ direction of fit: the world is one way, and it is the job of (that state of) the mind to conform to the world.^20^ Beliefs, therefore, have truth conditions. Desires, in contrast, have ‘mind-to-world’ direction of fit. Desires have content, however, the content of a desire does not represent the world as it is: it ‘describes’ the world as the agent wants it to be. Desires, thus, have ‘success’ rather than ‘truth’ conditions. Yet a prediction is clearly something that can come true or be false: it is true if it matches how the world will be or ends up being. Thus, if the ‘direction of fit’ picture is correct, desires cannot be the kind of mental state that represents the world. Consequently, desires cannot misrepresent the world since they do not represent the world.

The direction of fit picture has been compellingly called into question generally speaking (Frost 2014), but I want to focus on a worry more specific to desire. Nanay (2023) distinguishes between intrinsic and extrinsic direction of fit. Intrinsic direction of fit “is about the representation relation itself” (Ibid., 194) whereas extrinsic direction of fit “is not not a matter of the representation relation itself, but rather of how the representation is used or what functional role it plays” (Ibid., 195).

The issue with the classic direction of fit account is that it conflates the intrinsic and extrinsic notions. That is, it takes the difference between the content of belief and desires to be about intrinsic direction of fit when it should be about extrinsic. Nanay (Ibid., 253) argues that all “representations only represent descriptively, that is, … they can only have [world-to-mind] intrinsic direction of fit” but that even if this is the case, “all the options about extrinsic directions of fit are still very much open.” I am sympathetic to this claim, but it is stronger than the one I need. For Nanay, the world-to-mind intrinsic direction of fit is true for *all* representations, I only need it to be true for the representations involved in beliefs and desires, which I will now argue is the case.

Intrinsic direction of fit is a matter of how one would specify the content of a belief or desire that p. Suppose I desire that it is sunny in Boston tomorrow. Here, the content of the desire is clearly a proposition, and, *qua* proposition, it has truth conditions defined in relation to a state of affairs. This is, I take it, why Nanay argues that the representation has an intrinsic world-to-mind direction of fit.

Suppose, now, that I believe it will be sunny tomorrow, and it turns out to be overcast. Suppose, even, that I both believe and desire it. What should I *do* with those states? Regarding the belief, I should maintain or affirm it if I wake up and the sun is shining into my window, and I should revise it if I wake up and see nothing but shades of grey. Regarding the desire: if I had access to a weather-controlling machine, I should use it to make sure it is sunny tomorrow.^21^ Given that I sadly do not, I should be happy if the sun does shine tomorrow, and sad if it doesn’t (given my belief and desire in combination, I should be disappointed, that is, sad in a way that the reality is worse than my expectations). In other words, I should make my beliefs fit the world, and, if I can, and all else is equal (see note 14), try to make the world match my desire. The difference that Searle initially pointed out is best understood as a difference of what I, as an agent, should do with my various mental states—it is a difference of extrinsic direction of fit.

That beliefs and desires have the same intrinsic, but opposite extrinsic fits squares nicely with another piece of Arpaly and Schroeder’s view. As Schroeder (2004, 133) points out: desires get their representational content from “perceptual or cognitive representations … or more carefully, these representational capacities.” These representational capacities are also responsible for the content of beliefs: this is why, per Schroeder, “the possible contents of my perceptions and beliefs are identical to the possible contents of my desires” (Ibid). Thus, it is the very same representational capacities that give beliefs and desires their contents!

Given Nanay’s point about intrinsic versus extrinsic direction of fit, Arpaly and Schroeder’s claim that desires cannot be identified with predictions loses its bite. Desires can have the representational content of a prediction: we just shouldn’t do with them what we do with predictive beliefs.

## Addiction, Moral Responsibility, and the Revised Reward Theory

Whether the original or Revised version of the reward theory is correct has implications beyond the philosophy of mind and psychology. Arpaly and Schroeder articulate views on an impressive variety of issues in moral psychology and ethics that (on those views) turn on the nature of desire. Here, I shall discuss one such issue. Arpaly and Schroeder present an argument that addiction mitigates ascriptions of blameworthiness. Their argument, while clever and impressive, very much depends on the details of desire in a way that puts the difference between the two views front and center.

On their overall view, whether someone is overall an apt target for praise or blame depends on whether the actions issue from desires that are themselves constitutive of good or ill will (Arpaly and Schroeder 2014, 162). The details of their view on good and ill will are not relevant to the points I am making here; what is relevant is that they take addiction to involve a motivational state that is not desire. Likewise, I remain neutral here on the merit of their theory of responsibility: what matters is that their argument turns on the details of desire.

Their claim is based on the fact that addiction affects the expected and not received reward stream (Berridge and Robinson 1998). The neural realizers of the reward system (on Arpaly and Schroeder’s view) use dopamine as their primary neurotransmitter, and drugs of abuse have the consequence of inundating the brain–-and thus the reward system—with dopamine. Berridge and Robinson (Ibid.) show that this inundation affects the neural realizers of the reward system such that the expected reward stream will always overestimate the amount of reward to be received from the addict’s drug of choice.^22^

The upshot for Arpaly and Schroeder is that, while addicts do have intrinsic desires for their substance(s) of choice, the “motivational force” behind their addictive behavior is “out of proportion to how much [the drug] is intrinsically desired” (Arpaly and Schroeder 2014, 288). Rather, an aberrant motivational force—instantiated by the expected reward stream—is “added to the force that is proper to the intrinsic desire” (Ibid.) to create the total motivational force behind addictive behavior. But since we should be judged, on their view, only on the content (and strength) of our intrinsic desires, this extra bit of motivational force ought not enter our judgments of blameworthiness. Indeed, compared to someone who had a similar overall motivational force, but for whom that source was entirely attributable to their desire, the blameworthiness of the addict is mitigated.

If the Revised view I am proposing is correct, however, this argument will not work. For on the Revised view, it is the expected reward system that is affected by chronic addiction, and states of the expected reward system constitute the desires themselves. There may well be (and likely are) other avenues for mitigating responsibility for addiction. But their particular argument does not succeed in doing so.

## Concluding Remarks

In this discussion, I have offered a novel theory of desire that is a variant of Arpaly and Schroeders Reward Theory. The difference between the two views amounts to the difference between whether we desire what we predict (in the right way) we will find rewarding (my view) or what we will ultimately find rewarding (theirs). I have argued—unsurprisingly—that my view is the correct one.

If there is a ‘moral of the story’ to glean about desire, I believe it harkens back to the initial motivation for views like the Reward Theory. As I discussed in the introduction, one major motivation was to capture the connection between desire and action while moving away from the simple standard theory of desire.

I believe that Arpaly and Schroeder moved *too far* away. In their discussion of addiction, they acknowledge that the occurrent counterparts of expected reward states have motivational power. Indeed, they are *the* motivationally efficacious state, for, as I have argued, an occurrent counterpart of received reward states would occur at the wrong time. However, instead of identifying these motivationally efficacious states with occurrent desires, Arpaly and Schroeder identify desires with a state that is a step removed from them. Indeed, they specifically wanted to define desires independently of their effects, while overlooking the plausible—and traditional functionalist—option of defining desires as the *typical* but not *necessary* causes of those effects.

There is also a moral to glean about natural kindism. Recall that natural kindism dictates that the properties of a mental kind not specified by its stereotype are to be determined by the properties of the natural kind with which it can be identified. I take the stereotype of desire not to specify whether we desire what we will ultimately find rewarding or what we predict (in the right way) we will find rewarding. Intuition, as suggested by the examples throughout this paper, is on the side of the latter, but in a way that is not necessarily decisive. Thus it becomes an empirical question which of these options best corresponds with desire, and an empirical question with philosophical implications like the one described here.

This is the power and promise of natural kindism: to provide a framework to systematically relate empirical details to philosophical issues.

## Footnotes

1 This paper will be neutral on whether the ‘objects’ of desire are objects or states of affairs.

2 I do not take Anscombe herself to be committed to the standard theory: she is merely saying that the most basic *indicator* of a desire is action.

3 So, arguably, are the family of ‘evaluative’ theories of desire such as those proposed by Oddie (2005) and Tenenbaum (2007).

4 ‘Reward’ here is understood in a technical sense, which I will define in more detail in the next section.

5 More precisely, they identify appetitive desires with the reward system and aversive desires with a ‘punishment’ system that runs in parallel (Arpaly and Schroeder 2014; 127). I gloss over this detail for expository purposes. What I say regarding the reward system and desires can be understood as holding for the reward system and appetitive desires, as well as for the punishment system and aversive desires.

6 It is fair to worry whether Arpaly and Schroeder’s theory could deal with a modified Radioman, whose reward system generates the right kind of signal for him to have desires, when he turns on radios, and nothing else. I am not pursuing that line of inquiry in this discussion, though I do so in ([REDACTED] in preparation).

7 I am taking some liberty here in interpreting the neuroscience. The data for this conclusion was offered by Berridge and Robinson, who have a different interpretation of the function of these components (Berridge 2012) and don’t take the relevant neurological structures to be computing expected and received reward. I am ‘translating’ their conclusion into an interpretation of the reward system along the lines of Wise (2004; 2009), which most closely matches Arpaly and Schroeder’s account.

8 One need not adopt a strict causal theory of meaning to advocate natural kindism. Indeed, the way Griffiths deploys Putnam’s notion of ‘stereotypes’ suggests his view is best classified as a causal-descriptive hybrid (Devitt and Sterelny 1998).

9 More precisely, the *referents* of the kind, e.g., ‘belief’, are identified with the referents of the relevant physical (for humans: neural) kind. The kind *terms*, while not strictly identical since they denote different concepts, are nonetheless more than just nomically co-referential in the relevant species(es): we are warranted in using them interchangeably.

10 Arpaly and Schroeder also, *pace* Griffiths and other natural kindists, commit to a metaphysical picture where a natural kind is not defined in terms of its causal role, but rather where the casual role serves as a ‘reference-fixer’ (as in Kripke 1972) to locate the natural kind then defined in some other way. In their words, “To treat intrinsic desire as a natural kind is to hold that intrinsic desires are the things, whatever they turn out to be … causing the actions, feelings, and cognitions that we associate with terms such as ‘desire’” (Arpaly and Schroeder 2014, 144). However, “then intrinsic desires need not be … *constituted* by any of their most familiar effects” (Ibid, emphasis added). I believe this move is intended to distance themselves from the action theory of desire: if so, I am not sure it is necessary. For many of the original functionalists (e.g., Putnam 1963; Lewis 1966) defined mental terms as the *typical* but not *necessary* causes of their stereotypical effects. Adjudicating between this metaphysical picture (which I admit I prefer) and Arpaly and Schroeder’s is a topic for its own discussion. For our purposes what is important is: both the original and Revised Reward Theories are compatible with either.

11 Schroeder (2004, 62) claims this technical sense is how Schultz et al. (1992) define reward. Regardless of whether that is right, there are other—in my opinion more plausible—definitions of reward in literature (e.g., Levy and Glimcher 2012); I do not, however, pursue this line further in the current discussion.

12 I am setting aside accounts like Armstrong’s (1968), which identify dispositions with their causal bases.

13 The Marge example was developed from one suggested to me by [REDACTED] in personal communication, who in turn credits it to a conversation he had with [REDACTED] and [REDACTED]. I am thankful to all of them.

14 I am thankful to [REDACTED] in personal communication for suggesting what might plausibly be Marge and Homer’s desires.

15 Predictive cues further out are more valuable *assuming equal certainty* of obtaining the reward. *Ceteris paribus,* the more time elapsed between a cue and what it predicts, the more can go wrong; this makes obtaining the reward less certain. Nevertheless, and insofar as reward value and uncertainty are represented distinctly (Schultz 2016), we can say that what the reward system aims for is cues that are both further out in the future and as certain as possible.

16 It is worth noting, that conditioned stimuli can *become* intrinsically desired; for discussion of how, see Railton (2017).

17 An analogous process happens with respect to overestimating reward value and extinction learning as discussed in Sect. 3.

18 However, I am not sure it is necessary to treat representational capacities as proper parts. It seems to me that a desire that P can get its content from (metaphorically) being a P-shaped keyhole. I remain noncommittal on this mereological issue.

19 The specific relationship Schroeder posits between affect and reward is that a change in expected reward from t_0_ to t_1_ is reflected in a change in hedonic or pleasure value. Thus, if an organism desires that P and a token of either P or some P-related cue, Q, appears in the environment, there will be a momentary increase in pleasure as the organism increases the amount of expected reward it is about to receive. It is (in part) in this way that the RTD handles seemingly non-pleasure related desires, like the desire to do one’s duty. All the RTD implies is that one will feel a bit better when signs that one’s duty is being fulfilled present themselves.

20 The terms ‘mind-to-world’ and ‘world-to-mind’ are each used both ways: in two sources I am using (Frost 2014; Nanay 2023) they are used the opposite way! I follow Frost’s usage here.

21 As Frost (2014, 433) points out, the sense of ‘should’ is to be understood thinly: “There is certainly no moral sense in which the world should be changed to fit someone’s desire to murder out of mere curiosity.”.

22 I am again, per note 7, ‘translating’ Berridge and Robinson’s neuroscientific findings to the framework of Wise (2004; 2009).
